# The Effect of Prenatal and Neonatal Fluoride Exposure to Morphine-Induced Neuroinflammation

**DOI:** 10.3390/ijms25020826

**Published:** 2024-01-09

**Authors:** Patrycja Kupnicka, Joanna Listos, Maciej Tarnowski, Agnieszka Kolasa, Patrycja Kapczuk, Anna Surówka, Jakub Kwiatkowski, Kamil Janawa, Dariusz Chlubek, Irena Baranowska-Bosiacka

**Affiliations:** 1 Department of Biochemistry and Medical Chemistry, Pomeranian Medical University in Szczecin, Powstańców Wlkp. 72, 70-111 Szczecin, Poland; 2 Department of Pharmacology and Pharmacodynamics, Medical University of Lublin, Chodźki 4a, 20-093 Lublin, Poland; 3 Department of Physiology in Health Sciences, Pomeranian Medical University, 70-210 Szczecin, Poland; 4 Department of Histology and Embryology, Pomeranian Medical University, Powstańców Wlkp. 72, 70-111 Szczecin, Poland; 5 Department of Plastic, Endocrine and General Surgery, Pomeranian Medical University, 72-010 Szczecin, Poland

**Keywords:** morphine, fluoride, neuroinflammation, environmental exposure

## Abstract

Physical dependence is associated with the formation of neuroadaptive changes in the central nervous system (CNS), both at the molecular and cellular levels. Various studies have demonstrated the immunomodulatory and proinflammatory properties of morphine. The resulting neuroinflammation in drug dependence exacerbates substance abuse-related behaviors and increases morphine tolerance. Studies prove that fluoride exposure may also contribute to the development of neuroinflammation and neurodegenerative changes. Morphine addiction is a major social problem. Neuroinflammation increases tolerance to morphine, and neurodegenerative effects caused by fluoride in structures related to the development of dependence may impair the functioning of neuronal pathways, change the concentration of neurotransmitters, and cause memory and learning disorders, which implies this element influences the development of dependence. Therefore, our study aimed to evaluate the inflammatory state of selected brain structures in morphine-dependent rats pre-exposed to fluoride, including changes in cyclooxygenase-1 (COX-1) and cyclooxygenase-2 (COX-2) expression as well as microglial and astroglial activity via the evaluation of Iba1 and GFAP expression. We provide evidence that both morphine administration and fluoride exposure have an impact on the inflammatory response by altering the expression of COX-1, COX-2, ionized calcium-binding adapter molecule (Iba1), and glial fibrillary acidic protein (GFAP) in brain structures involved in dependence development, such as the prefrontal cortex, striatum, hippocampus, and cerebellum. We observed that the expression of COX-1 and COX-2 in morphine-dependent rats is influenced by prior fluoride exposure, and these changes vary depending on the specific brain region. Additionally, we observed active astrogliosis, as indicated by increased GFAP expression, in all brain structures of morphine-dependent rats, regardless of fluoride exposure. Furthermore, the effect of morphine on Iba1 expression varied across different brain regions, and fluoride pre-exposure may influence microglial activation. However, it remains unclear whether these changes are a result of the direct or indirect actions of morphine and fluoride on the factors analyzed.

## 1. Introduction

Opioid dependence is considered one of the most severe and common addictions [1,2], and the number of people with opioid use disorders is steadily increasing worldwide [3]. The development of this addiction is a result of the indirect action of morphine on dopamine receptors. First, the activation of opioid receptors causes a decrease in the activity of gamma-aminobutyric acid (GABA) receptors, which has an inhibitory effect on dopaminergic neurons and leads to dopamine release in structures associated with the development of addiction [4]. However, it is not only sudden changes in dopamine levels that contribute to the development of opioid addiction. Physical dependence is associated with the formation of neuroadaptive changes in the central nervous system (CNS), both at the molecular and cellular levels [3], depending on factors including the type of drug, method and timing of use, genetic predisposition, and exposure to environmental factors and the associated development of chronic low-grade inflammation, particularly in the central nervous system [5,6].

Various studies have demonstrated the immunomodulatory and proinflammatory properties of morphine [7,8]. Morphine-induced neuroinflammation appears to be particularly important not only in the mechanism of addiction [9,10], but also in morphine tolerance, by increasing neuronal excitability [11,12,13] and activating microglia and CNS astrocytes [14,15,16].

Fluoride is an abundant environmental agent with proven neurotoxic effects. It is naturally present in water and foods such as grains, fish, meat, milk, and tea [17,18,19], as well as in artificially enriched products such as fluoridated salt (0.25 mg fluoride per gram of salt) [17]. In many European countries, the level of fluoride in tap water must not be higher than 1.5 mg/L and is usually estimated to be between 0.3–0.7 mg/L [17], while a liter of mineral water may hold up to 5 mg of F^−^ [17,20]. The upper tolerable limit for fluoride intake is estimated to be 0.12 mg/kg/day (about 5 mg/d for children and 7 mg/d for adults) [21], whereas the level of 0.05 mg/kg/day (0.01 mg/kg/day for infants) might be considered an adequate intake level [17]. In non-endemic areas, daily intake in children and adults reaches an average of 3–4.4 mg/day [22]. However, in endemic areas, the level of fluoride intake might be on average 8.4 mg [23], 12.1 +/− 4.1 from water, and additionally 3.4 +/− 2.43 mg from diet [24] or even up to 14–19 mg daily in children and adults in high fluoride endemic areas, and 7.5–11 mg/L in medium endemic areas [25]. Because fluoride readily crosses the blood–brain barrier, chronic exposure to even low doses of this element leads to its accumulation in neural tissues and central nervous system dysfunction [26]. In the low-dose studies, 0.5 μmol/L (10 μg/L) was sufficient to induce lipid peroxidation and result in biochemical changes in brain cells, while 3 μmol/L (57 μg/L) induced inflammatory reactions in brain cells [27]. Although the mechanisms of fluoride neurotoxicity are still not well understood, it is known that the element contributes to the induction of oxidative stress and leads to neuronal apoptosis [26,28]. It may contribute to the development of neuroinflammation and neurodegenerative changes in the striata, motor cortices, cerebella, and amygdalae of rats [26,29,30,31]. 

The development of inflammation is due in part to the activation of cyclooxygenases (COX) 1 and 2, enzymes that catalyze the committed step in the formation of prostanoids such as thromboxane A2 and prostaglandin H2 from arachidonic acid [32]. Their expression may be constitutive or induced via tissue injury and inflammation. COX-1 is a housekeeping enzyme characterized by constitutive expression in all tissues. Glial COX-2 appears to play an important role in the development of neuroinflammation, and its expression is necessary to enter the resolution phase [33]. Inflammatory mediators may also include ionized calcium-binding adapter molecule 1 (Iba1), an indicator of activated macrophages that is uniquely expressed by microglia in the brain [34,35]; and glial fibrillary acidic protein (GFAP), expressed by astrocytes, cells that form glial scars after injury [36].

Both fluoride and morphine exhibit neurotoxic and neurodegenerative effects, including through the development of inflammation. Morphine addiction is currently a major social problem. Neuroinflammation increases tolerance to morphine and neurodegenerative effects caused by fluoride in structures related to the development of dependence may impair the functioning of neuronal pathways, change the concentration of neurotransmitters, and also cause memory and learning disorders [3,9,13]. Currently, there is no information in the literature regarding the impact of fluoride-induced inflammation in pre- and neonatal period on the development of morphine dependence. The neurodegenerative and neuroinflammatory properties of this element affecting the mesolimbic and mesocortical systems suggest that this element may influence the development of dependence [27,29,31]. Our study aims to evaluate the inflammatory state of selected brain structures in morphine-dependent rats pre-exposed to fluoride. Changes in COX-1 and COX-2 expression as well as microglial and astroglial activity are characteristic features of neuroinflammation [37]. Therefore, we analyzed the expression levels of inflammatory markers, i.e., COX-1 and COX-2, Iba1, and GFAP. The resulting neuroinflammation in drug dependence exacerbates substance abuse-related behaviors and increases morphine tolerance. However, it is not clear whether ongoing inflammatory processes in the brain can modulate the response to morphine.

## 2. Results

### 2.1. Gene Expression—rT-PCR Analysis

#### 2.1.1. COX-1

**Figure 1:**
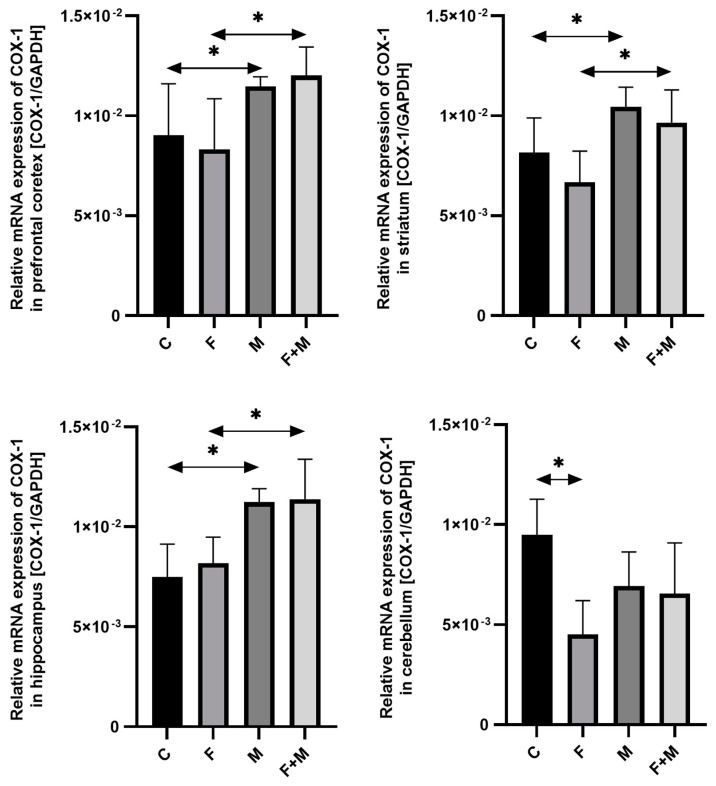
The relative mRNA expression of *COX-1* in the prefrontal cortices, striata, hippocampi, and cerebella of the rat brains in the control (C), fluoride (F), morphine (M), and fluoride + morphine (M + F) groups. The results are presented as means ± SD. The analysis was performed for 6 samples from each group. The statistical analysis was performed using Kruskal–Wallis one-way ANOVA, * *p* < 0.05.

In the prefrontal cortex, morphine administration and further withdrawal increased *COX-1* mRNA expression in the groups pre-exposed to fluoride (vs the group exposed only to F) and nonexposed to fluoride (vs the control group C), by 44% and 27%, respectively (*p* < 0.05). Also in the striatum, the groups exposed to morphine and to morphine and fluoride showed increased levels of *COX-1* by 28% and 55%, respectively, which was statistically significant for M vs. C and MF vs. F (*p* < 0.05). Similar changes were observed in the hippocampal structure, where mRNA expression increased by 39% and 50%, respectively (*p* < 0.05). The changes observed in the cerebellum were different from the other structures. The study groups were characterized by a significant decrease in *COX-1* mRNA expression, F by 52%, M by 27% (*p* < 0.05). However, morphine administration and further withdrawal did not alter *COX-1* expression in the fluoride pre-exposed groups. There was no change between M and MF (Figure 1).

#### 2.1.2. COX-2

**Figure 2:**
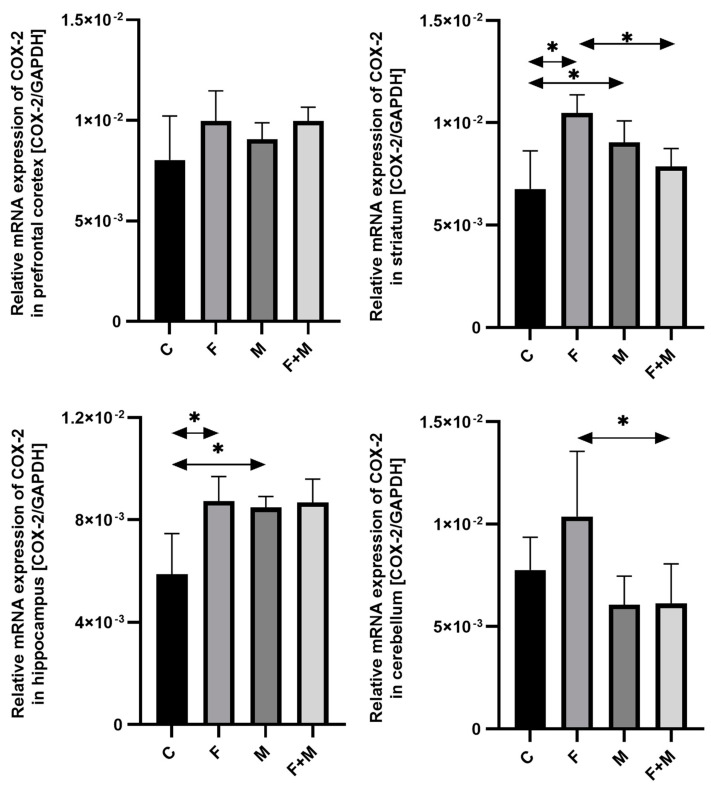
The relative mRNA expression of *COX-2* in the prefrontal cortices, striata, hippocampi, and cerebella of the rat brains in the control (C), fluoride (F), morphine (M), and fluoride + morphine (M + F) groups. The results are presented as means ± SD. The analysis was performed for 6 samples from each group. The statistical analysis was performed using Kruskal–Wallis one-way ANOVA, * *p* < 0.05.

The mRNA expression of *COX-2* in the prefrontal cortex was increased in all groups examined, but this difference was not statistically significant. However, in the striatum and hippocampus, there was a statistically significant increase. Exposure to fluoride resulted in a 55% increase in *COX-2* expression in the striatum and a 48% increase in the hippocampus compared to the control group (*p* < 0.05). When naloxone was administered to morphine-dependent rats, there was a 34% increase in *COX-2* expression in the striatum and a 45% increase in the hippocampus (*p* < 0.05). Furthermore, in the striatum and cerebellum, morphine withdrawal in rats pre-exposed to fluoride altered the mRNA expression of *COX-2* (compared to fluoride exposure), leading to a decrease of 25% and 41%, respectively (*p* < 0.05). There were no significant differences between the morphine group and the morphine-fluoride group (Figure 2).

#### 2.1.3. GFAP

**Figure 3:**
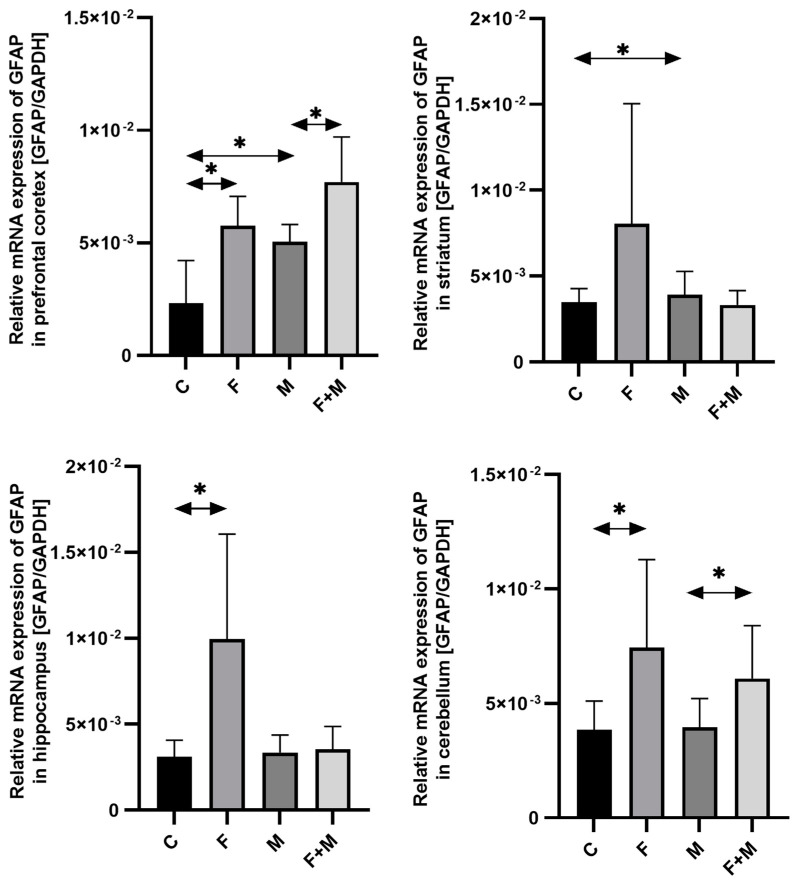
The relative mRNA expression of *GFAP* in the prefrontal cortices, striata, hippocampi, and cerebella of the rat brains in the control (C), fluoride (F), morphine (M), and fluoride + morphine (M + F) groups. The results are presented as means ± SD. The analysis was performed for 6 samples from each group. The statistical analysis was performed using Kruskal–Wallis one-way ANOVA, * *p* < 0.05.

In the prefrontal cortex, striatum, hippocampus, and cerebellum, fluoride exposure resulted in a significant upregulation of *GFAP* mRNA expression compared to the control group, with increases of 148%, 132%, 219%, and 93%, respectively (*p* < 0.05). Morphine withdrawal also led to an upregulation of *GFAP* expression, with increases of 117% in the prefrontal cortex and 12% in the striatum (*p* < 0.05). Furthermore, in the prefrontal cortex and cerebellum, pre-exposure to fluoride altered the response to morphine dependence (compared to fluoride exposure alone), resulting in additional increases in *GFAP* mRNA expression of 33% and 54%, respectively (*p* < 0.05) (Figure 3).

#### 2.1.4. Iba1

**Figure 4:**
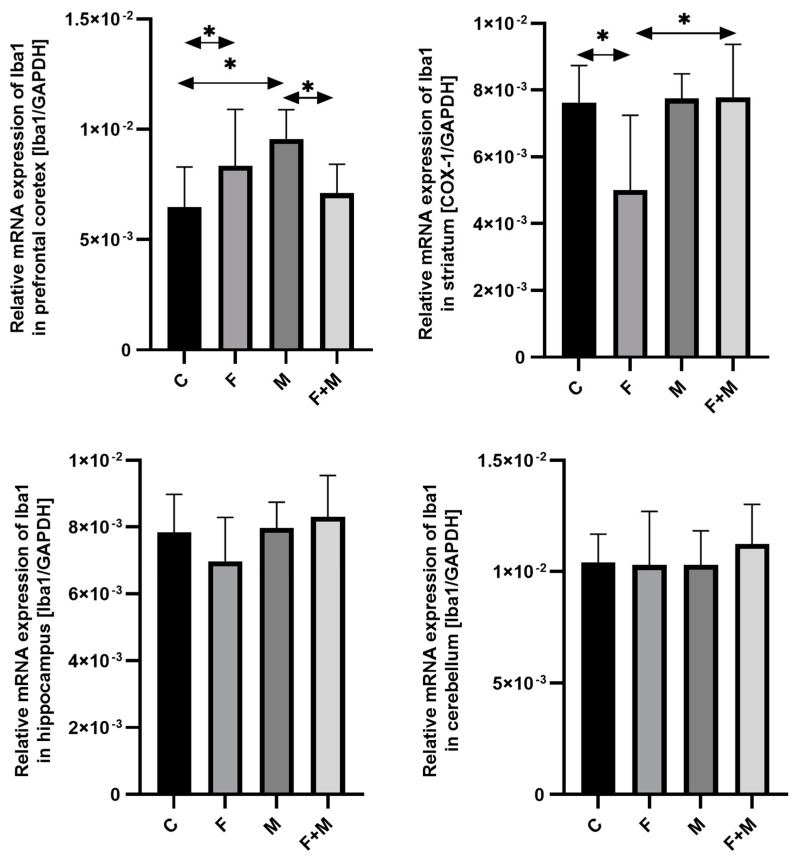
The relative mRNA expression of *Iba1* in the prefrontal cortices, striata, hippocampi, and cerebella of the rat brains in the control (C), fluoride (F), morphine (M), and fluoride + morphine (M + F) groups. The results are presented as means ± SD. The analysis was performed for 6 samples from each group. The statistical analysis was performed using Kruskal–Wallis one-way ANOVA, * *p* < 0.05.

In the prefrontal cortex, fluoride exposure increased the expression of *Iba1* by 29% compared to the control group (*p* < 0.05), while morphine dependence in the withdrawal model increased it by 48% compared to the control group (*p* < 0.05). Additionally, pre-exposure to fluoride altered the response to morphine withdrawal, leading to a downregulation of *Iba1* expression, with a 26% decrease compared to the morphine group (*p* < 0.05) (Figure 4). In the striatum, the expression of *Iba1* was significantly lower in the fluoride-exposed group compared to the control group (*p* < 0.05), and morphine dependence increased it by 55% compared to the fluoride-exposed group (*p* < 0.05). However, no significant changes were observed in the hippocampus and cerebellum (Figure 4).

Raw mean and SD values of the mRNA expression are given in the supplementary file (Table S1).

### 2.2. Protein Expression—Western Blot Analysis

#### 2.2.1. COX-1

**Figure 5:**
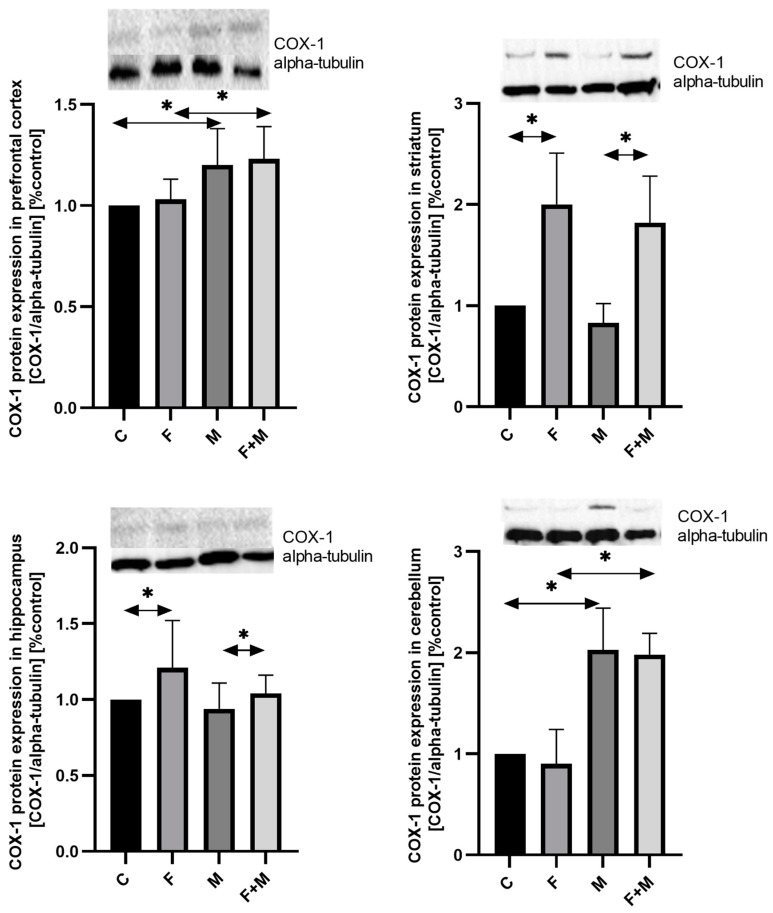
Representative Western blots and densitometric analysis of COX-1 protein expression levels (normalized to alpha-tubulin) in the prefrontal cortices, striata, hippocampi, and cerebella of rats from control (C), fluoride (F), morphine (M), and fluoride + morphine (M + F) groups. The results are expressed as means ± SD. The analysis was performed for 3 samples from each group. The statistical analysis was performed using Kruskal–Wallis one-way ANOVA, * *p* < 0.05.

In the prefrontal cortex, both morphine withdrawal in rats pre-exposed to fluoride (compared to fluoride exposure alone) and morphine withdrawal in rats not exposed to fluoride (compared to the control group) resulted in an upregulation of COX-1 protein expression by 23% and 20%, respectively (*p* < 0.05) (Figure 5).

In the striatum, the expression of COX-1 protein was significantly elevated by 2 times in the fluoride-exposed group compared to the control group (*p* < 0.05). This increase was also observed in the group of rats with both morphine dependence and fluoride exposure, with a notably higher elevation of 119% compared to the rats with morphine dependence alone (*p* < 0.05) (Figure 5).

Similar changes were observed in the hippocampus, where the fluoride-exposed group showed a 21% increase in COX-1 protein expression compared to the control group (*p* < 0.05). In the group of rats with both morphine dependence and fluoride exposure, there was an 11% increase compared to the rats with morphine dependence alone (*p* < 0.05) (Figure 5).

In the cerebellum, fluoride did not have an impact on the level of COX-1 expression. However, morphine increased the expression in both the morphine-dependent groups (M and MF) compared to the control group (*p* < 0.05) (Figure 5).

#### 2.2.2. COX-2

**Figure 6:**
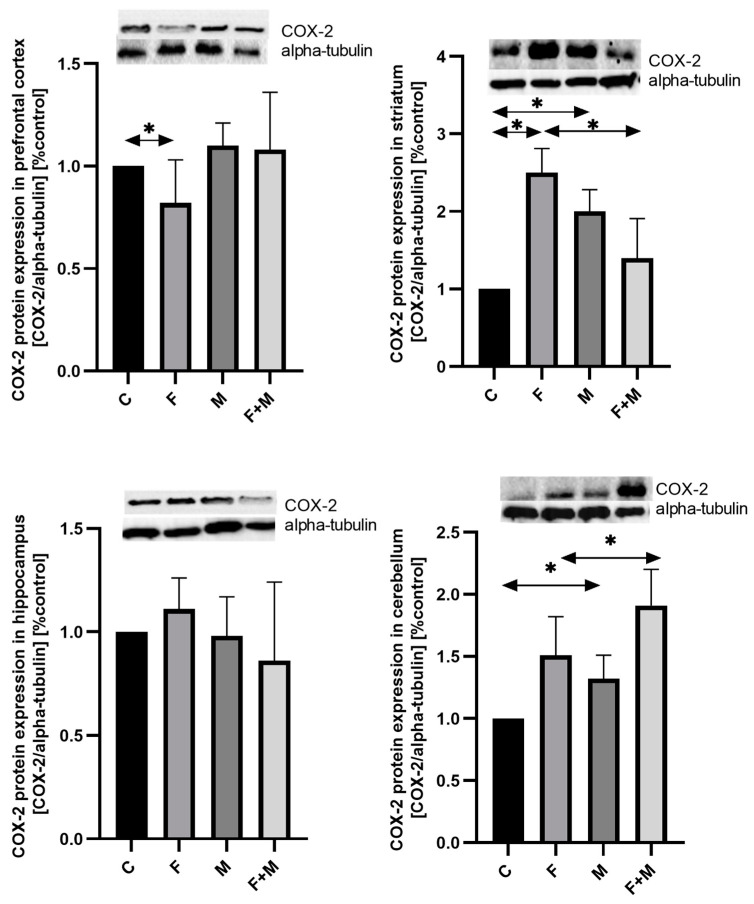
Representative Western blots and densitometric analysis of COX-2 protein expression levels (normalized to alpha-tubulin) in the prefrontal cortices, striata, hippocampi, and cerebella of rats from control (C), fluoride (F), morphine (M), and fluoride + morphine (M + F) groups. The results are expressed as means ± SD. The analysis was performed for 3 samples from each group. The statistical analysis was performed using Kruskal–Wallis one-way ANOVA, * *p* < 0.05.

In the prefrontal cortex, fluoride exposure resulted in the downregulation of COX-2 protein expression compared to the control group (*p* < 0.05) (Figure 6).

In the striatum, the fluoride-exposed group showed a 2.5-fold increase in the level of COX-2 protein expression compared to the control group, and morphine withdrawal led to a 2-fold increase compared to the control group (*p* < 0.05). However, in the group of rats with both morphine dependence and fluoride exposure (MF group), there was a 44% decrease in COX-2 expression compared to the fluoride-exposed group (*p* < 0.05) (Figure 6).

In the cerebellum, fluoride exposure increased the level of COX-2 expression by 51%, although this change was not statistically significant. Additionally, morphine administration increased expression by 32% compared to the control group (*p* < 0.05), and in the MF group compared to the fluoride-exposed group, there was a 25% increase in COX-2 expression (*p* < 0.05). No significant changes were observed in the hippocampus (Figure 6).

#### 2.2.3. GFAP Protein Expression

**Figure 7:**
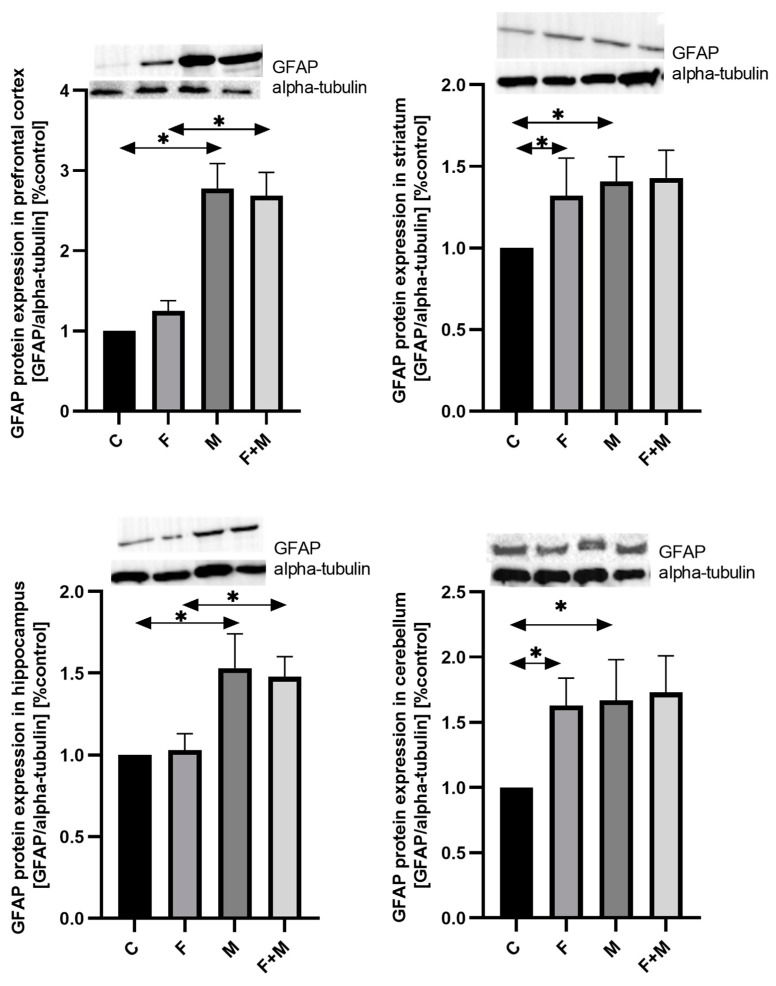
Representative Western blots and densitometric analysis of GFAP protein expression levels (normalized to alpha-tubulin) in the prefrontal cortices, striata, hippocampi, and cerebella of rats from control (C), fluoride (F), morphine (M), and fluoride + morphine (M + F) groups. The results are expressed as means ± SD. The analysis was performed for 3 samples from each group. The statistical analysis was performed using Kruskal–Wallis one-way ANOVA, * *p* < 0.05.

Morphine dependence resulted in an increase in GFAP protein expression in both the fluoride-exposed group (MF vs. F) and the non-exposed group (M vs. C) by 115% and 178%, respectively (*p* < 0.05). Similar changes were observed in other brain structures. However, in the striatum and cerebellum, fluoride exposure alone increased the expression of GFAP protein by 32% and 63%, respectively (*p* < 0.05). In the hippocampus, the increase in GFAP protein expression after morphine administration was 53%, while in the striatum and cerebellum, it was 41% and 67%, respectively (*p* < 0.05). Furthermore, in morphine-dependent rats pre-exposed to fluoride, there was a 44% increase in GFAP protein expression in the hippocampus (*p* < 0.05) (Figure 7).

#### 2.2.4. Iba1

**Figure 8:**
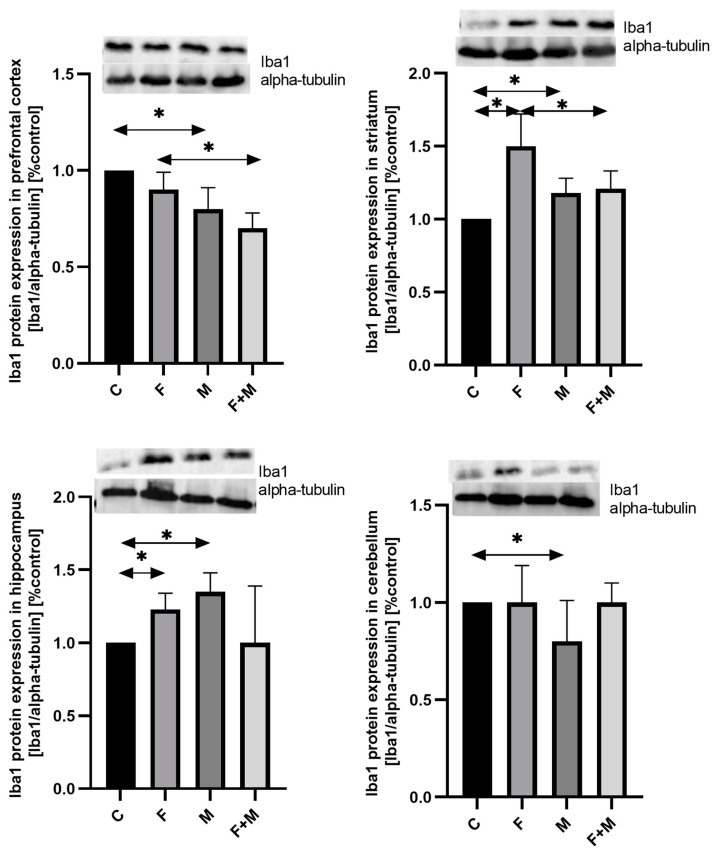
Representative Western blots and densitometric analysis of Iba1 protein expression levels (normalized to alpha-tubulin) in the prefrontal cortices, striata, hippocampi, and cerebella of rats from control (C), fluoride (F), morphine (M), and fluoride + morphine (M + F) groups. The results are expressed as means ± SD. The analysis was performed for 3 samples from each group. The statistical analysis was performed using Kruskal–Wallis one-way ANOVA, * *p* < 0.05.

In the prefrontal cortex, both morphine administration in rats pre-exposed to fluoride and those not exposed to fluoride resulted in a decrease in Iba1 protein expression by 22% and 20%, respectively (Figure 8).

In the striatum, fluoride exposure increased the expression of Iba1 protein by 50% compared to the control group (*p* < 0.05), while morphine administration increased it by 18% (*p* < 0.05). However, in the fluoride-exposed group with morphine administration (MF vs. F), there was a 19% decrease in Iba1 expression (*p* < 0.05) (Figure 8).

In the hippocampus, both morphine administration and fluoride exposure increased the level of Iba1 protein expression compared to the control group, with increases of 23% and 35%, respectively (*p* < 0.05). No significant change was observed between the fluoride-exposed and morphine-administered groups (Figure 8).

In the cerebellum, only morphine dependence had an impact on the level of Iba1 protein expression (*p* < 0.05), while no significant change was observed between the fluoride-exposed and morphine-administered groups (Figure 8).

Raw mean and SD values of the protein expression are given in the supplementary file (Table S2).

### 2.3. Protein Expression—Immunohistochemistry Analysis

#### 2.3.1. GFAP

**Figure 9:**
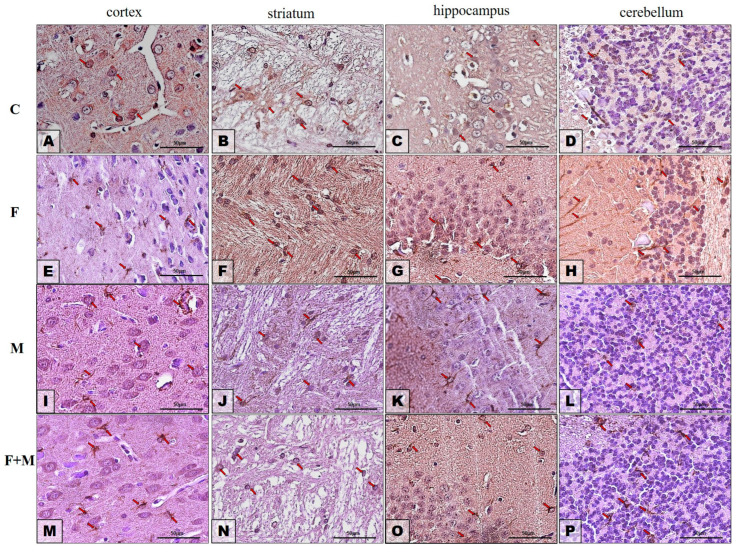
Representative microphotography showing immunoexpression of GFAP in the cortices (**A**,**E**,**I**,**M**), striata (**B**,**F**,**J**,**N**), hippocampi (**C**,**G**,**K**,**O**), and cerebella (**D**,**H**,**L**,**P**) of rats in the control (**A**–**D**), fluoride (**E**–**H**), morphine (**I**–**L**), and morphine and fluoride (**M**–**P**) groups. IHC reaction. Scale bar = 100 μm. Red arrows—immunopositive astrocytes or their processes.

Immunohistochemistry (IHC) analysis revealed that the immunoexpression of glial fibrillary acidic protein (GFAP) in the prefrontal cortex (neocortex) was highest in the brains of rats treated with both fluoride and morphine (Figure 9M, indicated by red arrows). In the control group, the expression of GFAP was the lowest (Figure 9A, red arrows), and the number of astrocytes positive for GFAP was quite similar in rats treated with fluoride (Figure 9E, red arrows) and morphine (Figure 9I, red arrows).

In the striatum region of the examined rat brains, there was nearly equal expression of GFAP in the control group, the fluoride-treated group, and the group treated with both fluoride and morphine (Figure 9B,J,N, red arrows). The striata of rats treated with fluoride alone exhibited the highest intensity of GFAP expression (Figure 9F, red arrows).

Figure 9C,G,K,O depict the GFAP reactivity in the hippocampus proper in all four studied groups (C, F, M, M + F). The level of GFAP reaction appeared to be very similar to that of the striatum, indicating that in the control group, fluoride-treated group, and fluoride plus morphine-treated group (Figure 9C,K,O, red arrows), the expression levels were quite equal. However, the GFAP expression was higher in the hippocampi from rats treated with fluoride alone (Figure 9G, red arrows).

In the cerebella of the studied animals (Figure 9D,H,L,P), GFAP immunoexpression exhibited similar patterns in the control group and morphine-treated group (Figure 9D,L, red arrows), as well as in the fluoride-treated group and the fluoride plus morphine-treated group (Figure 9H,P, red arrows). After exposure to fluoride and fluoride plus morphine, the GFAP expression was higher compared to the control and morphine-treated groups.

The results of the immunohistochemistry were consistent with the findings from Western blotting analysis.

#### 2.3.2. Iba1

The immunohistochemistry (IHC) analysis revealed differences in the immunoexpression of Iba1-positive microglia cells in different brain regions and treatment groups.

**Figure 10:**
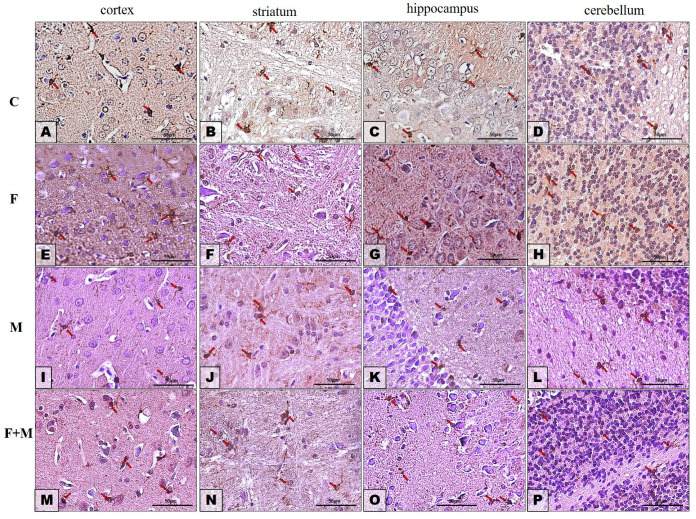
Representative microphotography showing immunoexpression of Iba1 in the cortices (**A**,**E**,**I**,**M**), striata (**B**,**F**,**J**,**N**), hippocampi (**C**,**G**,**K**,**O**) and cerebella (**D**,**H**,**L**,**P**) in rats from the control (**A**–**D**), fluoride (**E**–**H**), morphine (**I**–**L**) and morphine and fluoride (**M**–**P**) groups. IHC reaction. Scale bar = 100 μm. Red arrows show microglia cells.

In their prefrontal cortices (neocortices), control rats showed low immunoexpression of Iba1-positive microglia cells (Figure 10A, red arrows), while rats treated with fluoride plus morphine exhibited higher expression (Figure 10M, red arrows). The expression levels increased further in rats treated with fluoride alone (Figure 10F, red arrows), and the highest expression was observed in rats treated with morphine alone (Figure 10I, red arrows).

In the striatum, the intensity of Iba1-immunoreactivity was comparable among the control group (Figure 10B, red arrows), the morphine-treated group (Figure 10J, red arrows), and the fluoride plus morphine-treated group (Figure 10N, red arrows). The lowest immunoreactivity for Iba was observed in the striatum of fluoride-treated rats (Figure 10F, red arrows).

The immunoreactivity for Iba1 in the hippocampus proper appeared to be relatively equal among all studied groups of rats (Figure 10C,G,K,O, red arrows), although the lowest immunoreactivity was observed after fluoride treatment (Figure 10G, red arrows).

In the cerebellum, there were no significant differences in Iba1 expression among all studied groups of rats (Figure 10D,H,L,P, red arrows).

The results of immunohistochemistry were in line with Western Blotting. Table 1 presents summarized results of the IHC analysis.

## 3. Discussion

The development of addiction is influenced by several factors, including genetic predisposition, age, duration and type of drug use, and exposure to environmental factors. Neurobiological pathways and processes, such as the modulation of monoamine release, oxidative status, and inflammatory processes, play a role in addiction development [3,38,39]. Previous studies investigating the impact of environmental factors such as lead, using the morphine withdrawal model, have shown that rats pre- and neonatally exposed to lead exhibit increased morphine tolerance. This effect is associated with neuroadaptive changes in dopamine D2 and adenosine A1 receptors in the mesocortical limbic system [37,39], as well as neuroinflammatory processes in the central nervous system [37]. In our study, we aimed to examine the role of another environmental factor, fluoride, in this phenomenon. To achieve this, we used a model previously proposed by Kobayashi et al. (2011) and Morales-González et al. (2010), which allowed us to replicate blood fluoride concentrations observed in humans environmentally exposed to this element [40,41]. Fluoride contributes to the development of neuroinflammation by increasing the expression of matrix metalloproteinase 9 (MMP9) and matrix metalloproteinase 2 (MMP2) in the striatum, prefrontal cortex, and cerebellum [42]. These enzymes are involved in tissue remodeling and regenerative processes in response to elevated levels of pro-inflammatory cytokines. Furthermore, fluoride activates microglia [26,29], leading to the increased synthesis of pro-inflammatory cytokines such as interleukin-1 beta (IL-1β), interleukin 6 (IL-6), and tumor necrosis factor alpha (TNF-alpha) [43,44].

Previous studies have demonstrated that fluoride has an impact on morphine dependence and tolerance by influencing the levels and metabolism of dopamine in structures associated with dependence, as well as serotonin and norepinephrine in the hippocampus and neocortex. Additionally, it can modulate the expression of receptors for these monoamines [45,46]. This evidence highlights the potential of fluoride, as an environmental factor, to affect the state of dependence.

In our study, we confirmed that morphine dependence in the withdrawal model leads to changes in COX-1 and COX-2 expression in the analyzed tissues, contributing to microglia activation and astrogliosis. Furthermore, we observed that fluoride may contribute to the development of neuroinflammation by increasing the expression of COX-1 and COX-2 in the striatum and hippocampus. It may also impact microglia activation (as indicated by increased Iba1 expression) in the prefrontal cortex, striatum, and hippocampus, as well as trigger reactive astrogliosis (as indicated by increased GFAP expression) in the prefrontal cortex, striatum, hippocampus, and cerebellum at the mRNA level, and in the striatum and cerebellum at the protein level. These changes confirm that inflammatory response is involved in morphine withdrawal in dependent rats. 

### 3.1. COX-1 Dependent Inflammatory Response in Morphine Dependence Is Influenced by Perinatal Fluoride Exposure 

Morphine withdrawal induced the expression of COX-1 in the prefrontal cortex (mRNA and protein), striatum (mRNA), hippocampus (mRNA), and cerebellum (protein). Similar changes were observed in the MF group, where the levels of COX-1 were significantly higher in the analyzed brain regions. COX-1 is known to be overexpressed by microglia, endothelial cells, and perivascular cells during neuroinflammation in animals [47,48].

The upregulation of COX-1 leads to increased production of prostaglandins, such as PgE2 and PgD2, which can have detrimental effects on the formation of amyloid-β plaques, neuronal loss, and cognitive functioning. COX-1 appears to play a more significant role in neuroinflammation than COX-2 [48], and its upregulation is required for the overexpression of COX-2 [47]. Our analysis suggests that fluoride pre-exposure influences the response to morphine administration in the hippocampus. Previous studies have shown that in rats perinatally treated with fluoride, morphine can decrease the expression of D1 and D2 receptors, as well as dopamine concentration in the hippocampus [45]. The impulses originating from hippocampal areas may stimulate the mesolimbic pathway [3,49], which is crucial in the initiation and development of dependence [3]. Dopaminergic projections from the ventral tegmental area (VTA) to the hippocampus and vice versa can induce long-term potentiation (LTP) in the hippocampus in response to novel stimuli in rodents [50,51,52,53]. Furthermore, fluoride pre-exposure also modulates morphine withdrawal in the cerebellum. Cerebellar neurons reach neuronal groups that are important in motivation and learning, including those involved in the development of addiction, such as the VTA, striatum, prefrontal cortex, amygdala, hippocampus, and locus coeruleus [54,55,56,57,58,59,60]. Carta et al. demonstrated the existence of a direct excitatory pathway from the cerebellum to the VTA, the stimulation of which leads to dopamine neuronal activation [61]. Therefore, it has been suggested that in addition to disruption in corticostriatal–limbic loops, addiction is also associated with disruptions in cerebellar processing and connectivity [52,62]. The results presented in this study indicate that fluoride affects morphine-induced inflammatory processes, particularly in the hippocampus and cerebellum, and the changes in dopaminergic transmission caused by morphine [45] may be partially attributed to the increased inflammatory status.

### 3.2. COX-2 Expression Changes May Affect Response to Morphine, thus Modulating Dependence and Tolerance to Morphine in Perinatal Fluoride Exposure 

Morphine withdrawal also influenced the expression of COX-2. The substance of abuse itself increased the expression of COX-2 protein and/or mRNA in the striatum, hippocampus, and cerebellum. However, the expression in the striatum was lower compared to the fluoride group, suggesting that perinatal fluoride exposure influences the inflammatory response in morphine dependence. In the cerebellum, an increase in COX-2 protein level was observed, indicating an ongoing inflammatory process. However, withdrawal from morphine in the fluoride plus morphine group led to a decrease in COX-2 mRNA expression in this brain region.

COX-2 is primarily an inducible isoform and is considered a major contributor to the development of inflammation [4]. Its expression and activity are modulated by inflammatory signals, and it plays a crucial role as a source of prostaglandins in chronic inflammation [63,64]. In the central nervous system (CNS), both COX-1 and COX-2 are constitutively expressed. COX-2, in particular, plays a significant role in synaptic activity, long-term potentiation, long-term depression, memory consolidation, and neurovascular coupling during functional hyperemia [48,65]. It also contributes to the oxygenation of endocannabinoids, which represents an important metabolic pathway in neurons for regulating excitatory synaptic transmission [66]. 

Morphine withdrawal is known to decrease dopamine synthesis or increase its catabolism in the striatum, as well as decrease dopamine receptor expression [45]. Therefore, changes in the expression of COX-2 in the striatum, a major component of the reward system, and the cerebellum in the morphine withdrawal model, both in rats non-exposed and pre-exposed to fluoride, may affect the response to morphine, thus modulating dependence and tolerance.

Furthermore, COX-2 activity is necessary to initiate the resolution phase of inflammation [67], and it has been demonstrated that COX-2 inhibitors, such as celecoxib, can prevent undesirable effects of morphine, such as increased tumor growth and metastasis [68]. The decrease in COX-2 expression observed in the striatum in the fluoride plus morphine group may therefore impede the anti-inflammatory and neuroadaptive processes, potentially worsening the patient’s condition [48].

Our findings indicate that the expression of both COX-1 and COX-2 in morphine-dependent rats is influenced by prior fluoride exposure. Importantly, these effects vary depending on the brain region, with notable impacts observed in the hippocampus, the striatum, and, intriguingly, the cerebellum.

### 3.3. GFAP Increased Expression Indicates Active Astrogliosis in Pre- and Neonatal Fluoride Exposure

In addition to modulating the expression of cyclooxygenases, both morphine dependence and fluoride exposure led to increased GFAP expression in all studied brain structures, indicating active astrogliosis. This increase was sustained in the fluoride plus morphine group in the prefrontal cortex and hippocampus. However, in the striatum and cerebellum, while there was an increase in GFAP protein and mRNA expression in the morphine group, no increase was observed in the fluoride plus morphine group, suggesting an influence of fluoride on the response to morphine.

The influence of morphine on toll-like receptors has been previously described, with toll-like receptor 4 (TLR4) playing a role and being located in microglia and astrocytes. Activation of the nuclear factor kappa-light-chain-enhancer of activated B cells (NFκB) pathway leads to the release of cytokines such as TNF-alpha, IL-1β, andIL-6 [12,69,70]. In our study, GFAP, a protein expressed by astrocytes and microglia, was upregulated, indicating ongoing inflammatory and reparative processes. Iba1, which was also elevated in our study, seems to be involved in these processes as well [71,72]. The increased level of GFAP, along with the upregulation of COX-1 expression, suggests the presence of active inflammatory and reparative processes [47].

Astrogliosis, while undoubtedly necessary for maintaining brain integrity and promoting revascularization for metabolic support of brain tissue, can also have detrimental effects. Cells within glial scars release various neurodevelopmental inhibitory molecules that hinder the complete physical and functional recovery of the central nervous system [72,73]. Glial cells are also involved in neuronal plasticity processes and may play a role in the development of morphine tolerance. Astroglial cells express opioid receptor μ3, which is coupled with nitric oxide (NO) release and contributes to tolerance development [74].

In our study, pre-exposure to fluoride increased GFAP mRNA expression after morphine withdrawal in the prefrontal cortex and cerebellum. As previously mentioned, these structures, and their functional correlations, are associated with addiction [62]. The absence of an increase in GFAP protein expression in the striatum and cerebellum suggests that fluoride influences morphine-induced astrogliosis. Altering the inflammatory status of these structures may impact dopaminergic neurotransmission [75] and subsequently affect the functioning of corticostriatal–limbic loops.

### 3.4. Morphine Treatment Causes Structure-Dependent Iba1 Expression Changes That Are Influenced by Fluoride Pre-Exposure 

Iba1 is predominantly found in activated macrophages and is also expressed by microglia cells [76]. Its level is increased during ongoing neuroinflammatory processes [77]. Neuroinflammation is believed to contribute to neural adaptations following chronic exposure to drugs of abuse [10,78,79]. Our study demonstrates that morphine dependence and/or fluoride exposure affect the response of microglia (as indicated by Iba1 expression) in a structure-dependent manner.

The immunomodulatory effects of morphine have been extensively studied [80,81,82]. Recent research has shown that morphine not only contributes to the development of inflammation [9,83], but also leads to microglial immunosuppression by activating insufficient mitophagy via NLRX1 and through the differential regulation of toll-like receptors (TLRs) and acetylcholinesterase (AChE) by modulating P65 and TRAF6 [80,81]. In our study, downregulation of microglia activity (as indicated by decreased Iba1 protein expression) was observed in the prefrontal cortex and cerebellum. This suggests that neural adaptive mechanisms may have limited neuroinflammation [9]. The observed increase in Iba1 mRNA expression in the prefrontal cortex after withdrawal could be attributed to the compensatory response. Additionally, in the cerebellum, fluoride treatment prior to morphine dependence did not result in a decrease in Iba1 expression as observed in the non-fluoride-treated rats, indicating the influence of fluoride on morphine-induced immunosuppression in this brain region. Similar modulation of morphine response by fluoride was also observed in the hippocampus and striatum, where, despite increased Iba1 protein levels in morphine-dependent and/or fluoride-exposed rats, the expression of Iba1 remained unchanged in the fluoride plus morphine group. The ongoing inflammation after 2 months of fluoride exposure may have triggered neuroadaptive changes in these structures [9], as observed at the mRNA level in the striatum.

In vitro studies using microglial cultures have shown an increase in Iba1 expression after 6–48 h of morphine treatment [84]. However, both Iba1 and GFAP expression can be attenuated by the inhibition of P2X purinoceptor 4 (P2X4), which inhibits morphine-induced microglial migration and affects morphine tolerance [84,85]. In some cases, morphine administration itself can decrease the level of P2X4 in structures associated with the development of dependence, such as the striatum and prefrontal cortex [86]. Therefore, the results obtained in our study confirm the diverse effects of morphine on the inflammatory response and indicate that fluoride pre-exposure may influence this process.

Taken all together, we documented in that study that long-term perinatal exposure of rats to fluoride may influence an inflammatory response, and it may produce an effect on morphine withdrawal. Thus, we found another environmental factor which can take part in morphine dependence development and in morphine withdrawal. 

## 4. Materials and Methods

The study was approved by the local Ethics Committee of the Medical University in Lublin (No. 20/2014, approval date 10 November 2014) in accordance with the Directive 2010/63/EU on the protection of animals used for scientific purposes.

### 4.1. Animal Model

Wistar rats were used in this study. Parental generation (F0) was used to obtain the next male generation (F1). The mating process started for F0:Control (*n* = 4) and F0:Fluoride (*n* = 4) groups at the same time; subsequently, the age of the parental animals was comparable. The mating pairs were kept in separate cages. After one week, the females were separated from the males, and each pregnant female rat was placed in an individual cage. The control group received tap water (fluoride content 0.1–0.7 mg/L) for drinking, while the experimental group received water supplemented with sodium fluoride (50 mg/L) throughout the entire pregnancy. 

Females gave birth to 8 rats on average. Both females and males were used in the study. The fluoride supplementation continued until 21 days after birth, when the young rats were weaned from their mothers. The young rats from the experimental group continued to receive water with fluoride until 2 months of age. The control group had ad libitum access to tap water during the same period.

The rats drank approximately 31.1 mL (±4.2 mL) of fluoridated water daily, which equals 1.56 mg F-/rat/day. Based on previous reports [17,87,88], the dose of fluoride was adjusted to represent its concentration in the rat’s blood similar to that observed in the serum of people environmentally exposed (water, food) to fluoride (to achieve rat serum F- concentrations similar to those in humans, 5-fold higher doses should be administrated) compounds [17,40,41,42]. Previous studies showed that the level of serum fluoride in rats treated with 50 ppm F- water was 0.24 mg/L [19], and it did not differ from the level of F- in the serum of the control group [19,26]. The concentration of fluoride in people from endemic areas rages between 0.51–1.92 (average 1.07 +/− 0.3 mg/L) [89], and in patients with symptomatic fluorosis, the concentration was found to be 0.16–1.25 mg/L [90], when in healthy subjects the level reaches up to 0.045 mg/L [90].

**Figure 11:**
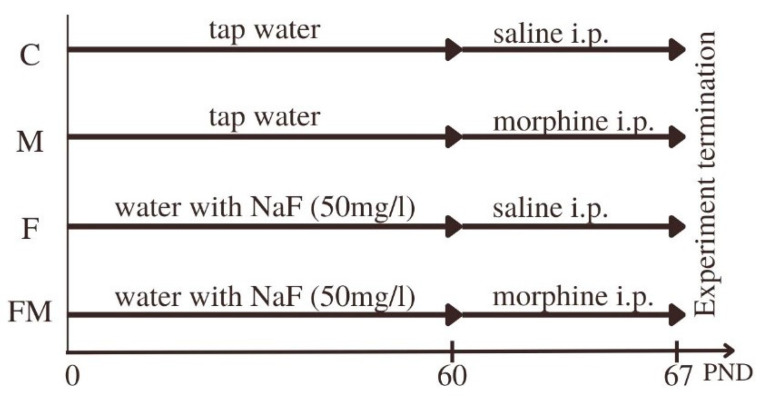
Division into groups included in molecular studies. C—Control group, M—Morphine-administered group, F—Fluoride-administered group, FM—Fluoride and morphine-administered group. PND—Postnatal days.

Starting from postnatal day 60 (PND 60), the animals were randomly divided into groups, and morphine dependence was induced by administering increasing doses of morphine (10.0, 15.0, 20.0, 25.0, 30.0, 35.0, 40.0, 50.0 mg/kg, intraperitoneal) twice a day for eight consecutive days (PND 60–67). On the following day (PND 68), a final dose of morphine (50.0 mg/kg) was administered, and one hour later, an opioid receptor antagonist, naloxone (2.0 mg/kg, intraperitoneal), was injected to induce morphine withdrawal (Figure 11). A saline+ naloxone group (rats receiving 0.9% NaCl and saline with naloxone (*n* = 6) on the last day of the experiment) was excluded from molecular studies. The rats were then decapitated, and their brains were quickly removed. The striata, hippocampi, prefrontal cortices, and cerebella were collected for neurochemical analysis and immediately frozen in liquid nitrogen. The tissues were stored at −80 °C until further analysis.

### 4.2. Gene Expression Analysis

Quantitative mRNA expression analysis of *COX-1*, *COX-2*, *Iba1*, and *GFAP* genes was performed using a two-step reverse transcription PCR method. The *GAPDH* gene was used as a reference gene for normalization. RNA was isolated from the tissue samples stored at −80 °C using the RNeasy MiniKit (Qiagen, Hilden, Germany) following the manufacturer’s instructions. The quality and quantity of RNA were determined using a NanoDrop ND 1000 (Thermo Fisher Scientific™, Waltham, MA, USA). The isolated RNA was then transcribed into cDNA using the Omniscript RT Kit (Qiagen, Hilden, Germany) according to the manufacturer’s instructions. 

Quantitative real-time PCR was conducted using a 7500 Fast Real-Time PCR System (Applied Biosystems, Foster City, CA, USA) and the Power SYBR Green PCR Master Mix (Applied Biosystems, Foster City, CA, USA) reagent. The real-time increase in the PCR reaction product was monitored through fluorescence measurement, which is proportional to the product’s concentration in the mixture. The mean from two measurements was used for further calculations. The ∆Ct relative quantification method was employed to calculate the values.

The following primer pairs were used for amplification:

− *GAPDH* forward: ATGACTCTACCCACGGCAAG, reverse: CTGGAAGATGGT GATGGGTT
− *Iba1* forward: GATTTGCAGGGAGGAAAAGCT, reverse: AACCCCAAGTTTCTCCAGCAT
− GFAP forward: GGTGGAGAGGGACAATCTCA, reverse: CCAGCTGCTCCTGGAGTTCT
− *COX-1* forward: GTTCACAGGAGAGAAGGAGATG, reverse: GGAGCCCCCATCTCTATCATGC
− *COX-2* forward: AATGAGTACCGCAAACGCTTCT, reverse: AGCCATTTCTTTCTCTCCTGTAAG.

### 4.3. Western Blot Analysis

The tissue samples from the striata, prefrontal cortices, hippocampi, and cerebella were treated with RIPA lysis buffer containing protease and phosphatase inhibitors (cOmplete™, Mini Protease Inhibitor Cocktail, Roche, Switzerland, PhosSTOP™, Roche, Switzerland). The protein concentration in the resulting filtrate was determined using the Pierce™ BCA Protein Assay Kit (Thermo Fisher Scientific™, Waltham, MA, USA).

For protein analysis, electrophoresis was performed under denaturing conditions using a 12% polyacrylamide gel (SDS-PAGE), with 30 µg of protein loaded into each well. The proteins were then transferred onto a 0.45 µm PVDF membrane (Thermo Fisher Scientific™, Waltham, MA, USA) using a wet transfer method. The membranes were subsequently incubated in a blocking buffer (5% milk) for 60 min.

The expression of COX-1 (ab81296), COX-2 (ab62331), Iba1 (ab178846), and GFAP (ab68428) proteins was detected using antibodies from Abcam (Cambridge, UK) at a dilution of 1:800. HRP-labeled secondary antibodies anti-mouse (ab6789) and anti-rabbit (ab205718) from Abcam (Cambridge, UK) were used. The expression of alpha-tubulin was detected using alpha-tubulin antibody (ab7291) from Abcam (Cambridge, UK).

The membranes were developed using an ECL Advance Western Blotting Detection Kit (GE Healthcare, Chicago, IL, USA), and the protein bands were visualized using the Molecular Imager ChemiDoc XRS+ (Bio-Rad, Hercules, CA, USA).

### 4.4. Immunohistochemical Analysis 

The dissected brain specimens were fixed in a 4% neutral buffered formalin solution for 24 h. Subsequently, they underwent a series of washes with distilled water, ethanol, and methanol to remove any residual fixative. The tissues were then dehydrated using a series of washes with absolute ethanol and xylene. After saturation with liquid paraffin, the samples were embedded in paraffin blocks.

Serial sections 3–5 µm in thickness were prepared from the paraffin-embedded tissues using a microtome (MICROM HM340E) and placed on silane-coated histological slides (3-aminopropyl-triethoxysilane, Thermo Scientific, Waltham, MA, USA). The sections were deparaffinized using xylene and ethanol in decreasing concentrations. 

To expose the epitopes, the deparaffinized sections were subjected to heat-induced antigen retrieval using a microwave oven in citrate buffer (pH 6.0). After cooling and washing with PBS, the sections were incubated with primary antibodies against the glial fibrillary acid protein (GFAP, a marker of astrocytes; Abcam, cat. no.: ab68428, diluted 1:250) and Iba1 (a marker of microglia cells; Abcam, cat. no.: ab178846, diluted 1:1000) for 60 min at room temperature. 

To visualize the antigen–antibody complex, the DAKO LSAB + System-HRP (DakoCytomation, cat. no.: K0679) was used, which employs the avidin–biotin–horseradish peroxidase reaction with diaminobenzidine (DAB) as the chromogen. The presence of brown pigmentation was microscopically observed (Leica DM5000 B, Leica Microsystems, Wetzlar, Germany) to determine positive staining.

### 4.5. Statistical Analysis

The statistical analysis was conducted using Statistica 13 software (StatSoft, Poland). The results are presented as mean values ± standard deviation (SD). The normality of the data distribution was assessed using the Shapiro–Wilk W test, which indicated a lack of conformity with the normal distribution. Therefore, Kruskal–Wallis one-way ANOVA was used to compare the groups. Statistical significance was defined as *p* < 0.05.

## 5. Conclusions

Our study provides evidence that both morphine administration and fluoride exposure have an impact on the inflammatory response by altering the expression of COX-1, COX-2, Iba1, and GFAP in brain structures involved in dependence development, such as the prefrontal cortex, striatum, hippocampus, and cerebellum. We observed that the expression of COX-1 and COX-2 in morphine-dependent rats is influenced by prior fluoride exposure, and these changes vary depending on the specific brain region, with the hippocampus, striatum, and cerebellum playing prominent roles. Additionally, we observed active astrogliosis, as indicated by increased GFAP expression, in all brain structures of morphine-dependent rats, regardless of fluoride exposure. Furthermore, the effect of morphine on Iba1 expression varied across different brain regions, and fluoride pre-exposure may influence microglial activation. However, it remains unclear whether these changes are a result of the direct or indirect actions of morphine and fluoride on the factors analyzed. Further in-depth studies are necessary to elucidate the underlying mechanisms responsible for the observed results.

## Figures and Tables

**Table 1:** Summary of the expression of Iba-1 and GFAP) in the control and study groups presented as intensity of immunostaining.

	Prefrontal Cortex		Striatum		Hippocampus		Cerebellum	
	Iba1	GFAP	Iba1	GFAP	Iba1	GFAP	Iba1	GFAP
Control (C)	+	+	++	+	+	+	+	+
Fluoride (F)	+	++	+++	+++	++	++	+	++
Morphine (M)	+++	++	++	+	+	+	+	+
Morphine + Fluoride (M + F)	++	+++	++	+	+	+	+	++

Intensity of immunostaining scored as weakly positive (+), moderately positive (++) or strongly positive (+++).

## Data Availability

Data available within the article or its supplementary materials.

## Supplementary Materials

The supporting information can be downloaded at: https://www.mdpi.com/article/10.3390/ijms25020826/s1
