# Supplementary material for: The Effect of Prenatal and Neonatal Fluoride Exposure to Morphine-Induced Neuroinflammation
Source: Int J Mol Sci. 2024 Jan 9;25(2):826. doi: 10.3390/ijms25020826 (PMC10815549; doi:10.3390/ijms25020826)
Supplement: Supplementary file 1 [file ijms-25-00826-s001.zip › ijms-2780613-supplementary.pdf]

Supplementary Materials

Table S1: Mean and SD values of the mRNA expression of COX-1, COX-2. Iba1 and GFAP in prefrontal cortex (PFC), striatum, hippocampus and cerebellum of each studied group.

		Control (C)	Fluoride (F)	Moprhine (M)	Fluoride+Morphine (MF)
**COX-1**	PFC	0.0090±0.0026	0.0083±0.0025	0.0115±0.0005	0.0120±0.0014
	Striatum	0.0082±0.0017	0.0067±0.0016	0.0104±0.0010	0.0097±0.0016
	Hippocampus	0.0075±0.0017	0.0082±0.0013	0.0112±0.0007	0.0114±0.0020
	Cerebellum	0.0095±0.0018	0.0045±0.0017	0.0069±0.0017	0.0066±0.0025
**COX-2**	PFC	0.0080±0.0021	0.0010±0.0015	0.0091±0.0008	0.0010±0.0007
	Striatum	0.0068±0.0019	0.0105±0.0009	0.0090±0.0011	0.0079±0.0009
	Hippocampus	0.0059±0.0016	0.0087±0.0009	0.0085±0.0004	0.0087±0.0009
	Cerebellum	0.0078±0.0016	0.0104±0.0032	0.0061±0.0014	0.0061±0.0019
**Iba1**	PFC	0.0065±0.0018	0.0083±0.0025	0.0096±0.0013	0.0071±0.0013
	Striatum	0.0076±0.0011	0.0050±0.0022	0.0077±0.0007	0.0078±0.0016
	Hippocampus	0.0078±0.0011	0.0070±0.0013	0.0080±0.0008	0.0083±0.0012
	Cerebellum	0.0104±0.0013	0.0103±0.0024	0.0103±0.0015	0.0112±0.0018
**GFAP**	PFC	0.0023±0.0019	0.0058±0.0013	0.0051±0.0008	0.0077±0.0020
	Striatum	0.0035±0.0008	0.0080±0.0070	0.0039±0.0014	0.0033±0.0008
	Hippocampus	0.0031±0.0010	0.0010±0.0061	0.0034±0.0010	0.0036±0.0013
	Cerebellum	0.0038±0.0013	0.0074±0.0038	0.0040±0.0013	0.0061±0.0023

Table S2: Mean and SD values of the protein expression of COX-1, COX-2. Iba1 and GFAP in Prefrontal cortex (PFC), striatum, hippocampus and cerebellum of each studied group.

		Control (C)	Fluoride (F)	Moprhine (M)	Fluoride+Morphine (MF)
**COX-1**	PFC	1.00±0.00	1.03±0.10	1.20±0.18	1.23±0.16
	Striatum	1.00±0.00	2.00±0.51	0.83±0.19	1.82±0.46
	Hippocampus	1.00±0.00	1.21±0.31	0.94±0.17	1.04±0.12
	Cerebellum	1.00±0.00	0.90±0.34	2.03±0.41	1.98±0.21
**COX-2**	PFC	1.00±0.00	0.82±0.21	1.1±0.11	1.08±0.28
	Striatum	1.00±0.00	2.50±0.31	2.00±0.28	1.40±0.51
	Hippocampus	1.00±0.00	1.10±0.15	0.98±0.19	0.86±0.38
	Cerebellum	1.00±0.00	1.51±0.31	1.32±0.19	1.91±0.29
**Iba1**	PFC	1.00±0.00	0.90±0.09	0.80±0.11	0.70±0.08
	Striatum	1.00±0.00	1.50±0.22	1.18±0.10	1.21±0.12
	Hippocampus	1.00±0.00	1.23±0.11	1.35±0.13	1.01±0.39
	Cerebellum	1.00±0.00	1.01±0.19	0.80±0.21	1.01±0.10
**GFAP**	PFC	1.00±0.00	1.25±0.13	2.78±0.31	2.69±0.29
	Striatum	1.00±0.00	1.32±0.23	1.41±0.15	1.43±0.17
	Hippocampus	1.00±0.00	1.03±0.10	1.53±0.21	1.48±0.12
	Cerebellum	1.00±0.00	1.63±0.21	1.67±0.31	1.73±0.28
